# Volumetric‐modulated arc therapy using multicriteria optimization for body and extremity sarcoma

**DOI:** 10.1120/jacmp.v17i6.6547

**Published:** 2016-11-08

**Authors:** Michael R. Young, David L. Craft, Caroline M. Colbert, Kyla Remillard, Liam Vanbenthuysen, Yi Wang

**Affiliations:** ^1^ Department of Radiation Oncology Massachusetts General Hospital and Harvard Medical School Boston MA USA; ^2^ Department of Biomedical Engineering and Biotechnology University of Massachusetts Intercampus MA USA; ^3^ Department of Nuclear Science and Engineering Massachusetts Institute of Technology Cambridge MA USA

**Keywords:** VMAT, MCO, sarcoma, treatment planning, dosimetric comparison, deliverability, quality assurance

## Abstract

This study evaluates the implementation of volumetric‐modulated arc therapy (VMAT) using multicriteria optimization (MCO) in the RayStation treatment planning system (TPS) for complex sites, namely extremity and body sarcoma. The VMAT‐MCO algorithm implemented in RayStation is newly developed and requires an integrated, comprehensive analysis of plan generation, delivery, and treatment efficiency. Ten patients previously treated by intensity‐modulated radiation therapy (IMRT) with MCO were randomly selected and replanned using VMAT‐MCO. The plan quality was compared using homogeneity index (HI) and conformity index (CI) of the planning target volume (PTV) and dose sparing of organs at risk (OARs). Given the diversity of the tumor location, the 10 plans did not have a common OAR except for skin. The skin D_50_ and $D_{\text{mean}}$ was directly compared between VMAT‐MCO and IMRT‐MCO. Additional OAR dose points were compared on a plan‐by‐plan basis. The treatment efficiency was compared using plan monitor units (MU) and net beam‐on time. Plan quality assurance was performed using the Sun Nuclear ArcCHECK phantom and a gamma criteria of 3%/3 mm. No statistically significant differences were found between VMAT‐ and IMRT‐MCO for HI and CI of the PTV or D_50_ and $D_{\text{mean}}$ to the skin. The VMAT‐MCO plans showed general improvements in sparing to OARs. The VMAT‐MCO plan set showed statistically significant improvements over the IMRT‐MCO set in treatment efficiency per plan MU (p<0.05) and net beam‐on time (p<0.01). The VMAT‐MCO plan deliverability was validated. Similar gamma passing rates were observed for the two modalities. This study verifies the suitability of VMAT‐MCO for sarcoma cancer and highlighted the comparability in plan quality and improvement in treatment efficiency offered by VMAT‐MCO as compared to IMRT‐MCO.

PACS number(s): separated by commas 87.55.D, 87.55.de, 87.55.Qr

## I. INTRODUCTION

Volumetric‐modulated arc therapy (VMAT) is the dynamic evolution of intensity‐modulated radiation therapy (IMRT). VMAT uses arcs to produce highly conformal dose distributions in complex patient geometry via concerted action of gantry rotation, multileaf collimator (MLC) motion, and dose rate modulation. Unlike the fixed gantry angles characteristic of IMRT delivery, VMAT involves dynamic gantry movement, which requires accurate synchronization of all moving components.^(1)^ The decreased treatment time, potential increased OAR dose sparing, and optimization of monitor units (MUs) afforded by VMAT over IMRT planning^(2)^ makes it ideal for clinical implementation.

VMAT planning is currently available in several commercial treatment planning systems (TPS), including Pinnacle SmartArc (Philips, Inc., Andover, MA), Monaco (Elekta, Inc., Stockholm, Sweden), Eclipse (Varian Medical Systems, Inc., Palo Alto, CA), and RayStation (RaySearch Laboratories, Stockholm, Sweden). Each system has its advantages and disadvantages for VMAT planning, and requires an expert planner in order to achieve high‐quality plans. In this study we focused on VMAT planning in RayStation, a TPS popularized by its development and implementation of multicriteria optimization (MCO). The MCO algorithm increases IMRT plan quality by allowing planners to better approach an optimal plan by interactively balancing several treatment objectives and constraints.^(3)^ Planners and physicians navigate high‐dimensional Pareto surfaces and weigh trade‐offs between objectives in real time to improve planning and plan quality. The mathematics and utility of MCO are elucidated in greater detail by Craft and Bortfeld^(4)^ and recent studies suggest dosimetric and efficiency advantages of MCO in generating optimal IMRT treatment plans.^(5)^

Algorithmic improvements in RayStation v4.7.2 have fully extended MCO to VMAT planning, but to date VMAT‐MCO has only been assessed and validated for prostate.^(6)^ We aim to implement fully‐functional VMAT‐MCO planning for more advanced treatment sites, and begin with sarcoma. Our integrative analysis includes an evaluation of sarcoma cancer VMAT‐MCO plan generation, delivery efficiency, and dosimetric comparison with IMRT‐MCO. The purposes of this study are twofold: to generate and deliver the first sarcoma cancer treatments using RayStation VMAT‐MCO, and subsequently to compare the generated plans’ dosimetric and delivery characteristics to paired IMRT‐MCO plans.

## II. MATERIALS AND METHODS

### A. Sarcoma cancer cases

Ten randomly‐selected sarcoma cases were planned with VMAT‐MCO by dosimetrists using patient CT scans and the target volumes for plans previously implemented clinically using IMRT‐MCO. Of the 10 plans used, 5 were for extremity sarcoma and 5 for body sarcoma patients. Sarcomas grow in connective tissue in various regions of the body, and thus planning target volumes (PTV), clinical target volumes (CTV), and their respective prescriptions varied by case. To account for the diversity of sarcoma locations, we compared treatments of the right arm, forearm, leg, gluteus, calf, left torso, and pelvis. Table 1 shows the target volumes and prescription for each plan. Sarcoma planning standards at our clinic require that 100% of the PTV receives 95% of the prescribed dose and that the maximum dose to the PTV is less than 110% of the prescribed dose. Each plan optimization used the same objectives and constraints as the corresponding IMRT‐MCO plan.

**Table 1:** Target volume and prescription dose for each selected sarcoma treatment

				*# of Fields*		*# of Control Points*	
*Plan #*	*Type*	*Target*	*Rx Dose (cGy)*	*IMRT*	*VMAT*	*IMRT*	*VMAT*
1	Extremity	Rt Calf	4400	7	4	138	189
2	Extremity	Rt Thigh	4400	5	4	100	126
3	Extremity	Rt Arm	5000	5	2	150	222
4	Extremity	Rt Forearm	6600	5	4	116	139
5	Extremity	Rt Leg	5000	6	4	164	169
6	Body	Lt Pelvis	6900	6	2	100	196
7	Body	Pelvis	5404	7	2	166	282
8	Body	Lt Torso	5000	8	2	116	216
9	Body	Rt Gluteus	5000	7	2	182	178
10	Body	Rt Gluteus	1600	6	2	194	178

### B. VMAT‐MCO

One goal of MCO is to allow for planners of any level to achieve high‐quality treatment plans in less time and with easier physician interaction than rival approaches. It allows for real‐time navigation of the optimization solution set, affording planners and physicians a valuable tool for finding and selecting Pareto‐optimal plans for treatment. The utility of MCO, and particularly IMRT‐MCO, compared to non‐MCO algorithms has been studied (e.g., McGarry et al.^(7)^ and Hong et al.^(8)^), but this comparison has not yet been fully extended to VMAT.

RayStation MCO uses a fluence‐based approach in its MCO module, meaning that the precomputed plans are optimized via fluence maps rather than the deliverable segment‐based dose engine. Computing a deliverable plan after navigation can lead to plan degradation. This has not proven to be a large problem clinically for MCO‐IMRT, but initial experience with MCO‐VMAT in RayStation v.4.0 showed that the discrepancy between navigated dose and final deliverable plan dose was often unacceptably large. This is because VMAT is a more challenging optimization problem due to its inherent nonconvexity.^(9)^ Clinical VMAT algorithms such as that implemented in RayStation also put a high focus on delivery efficiency, which can compromise plan quality, particularly for highly modulated fields. If they instead focused on fluence map reproduction fidelity, they could use sliding window‐like VMAT plans to reproduce optimal fluence maps. This would lead to longer plan delivery times, but would make MCO‐VMAT more similar to MCO‐IMRT, which achieves high fidelity in dose recreation by allowing for each fluence map to be recreated with many MLC segments.^(10)^

### C. Plan generation, delivery, and measurement

#### C.1 Plan generation

Plans in this study were generated with the RayStation v.4.7.2 VMAT‐MCO module. In order to help minimize study uncertainty, each VMAT‐MCO plan was generated by the dosimetrist who generated its corresponding IMRT‐MCO plan. IMRT‐MCO plans used between five and nine fields, depending on treatment site, and the number of fields and dose control points for each plan can be found in Table 1. VMAT‐MCO plans used two or four arcs, depending on treatment site, and the number of fields and dose control points for each plan can also be found in Table 1. All VMAT‐MCO plans were set at 2° gantry spacing at variable gantry speeds and dose rates. Each planner iteratively set constraints and fine‐tuned objectives for MCO optimization to approach an optimal treatment plan. Objectives and constraints varied for each plan, but generally sought maximal dose sparing to relevant organs at risk (OAR) and maximal target coverage to the PTV. Plans then underwent dose reconstruction using machine parameters, at which point each VMAT‐MCO plan was confirmed to be clinically viable by a physician.

#### C.2 Plan delivery

All 20 plans (10 VMAT and 10 IMRT) were delivered on an Elekta Agility linear accelerator (Elekta, Stockholm, Sweden) at an energy of 6 M V. The Elekta Agility was previously commissioned for both IMRT and VMAT^(11)^ treatments. Machine parameters include a maximum field size of $40\times 40\;{\text{cm}}^{2}$, a leaf pitch of 0.5 cm, and 160 MLC leaves that move with a maximum speed of 3.5 cm/s.^(12)^

#### C.3 Plan measurement

All 20 plans were measured using a Sun Nuclear ArcCHECK, a $21\times 21\times 15\;{\text{cm}}^{3}$ cylindrical dose measurement tool which consists of 1,386 0.019 mm^2^ diode detectors each spaced 1.0 cm apart.^(13)^ Prior studies have validated the ArcCHECK as an IMRT and VMAT quality assurance (QA) measuring tool (e.g., Nelms et al.^(14)^). For dosimetric evaluation, we use a gamma passing rate of 3%/3 mm.^(15)^ The ArcCHECK is a three‐dimensional measuring device which allows for entry and exit dose measurements to provide valuable information for VMAT QA potentially missed by two‐dimensional QA devices.^(16)^

### D. Plan comparison metrics

VMAT‐MCO and IMRT‐MCO plans were compared by plan quality and treatment efficiency. Plan quality was evaluated by comparing OAR dose sparing, conformity index (CI), and homogeneity index (HI) averages for the 10 patient sets. Due to the wide spread of targets among the selected sarcoma plans, only dose‐sparing comparisons of D_50_ and $D_{\text{mean}}$ to an external body contour of the skin were evaluated between modalities. To compensate for the wide range in prescription doses, skin doses for each plan were scaled to a prescription dose of 5000 cGy by a factor of 5000 cGy/RxDose. The variation in target by plan prevents further direct comparisons between the VMAT‐MCO and IMRT‐MCO sets. To compensate, dosimetrists also selected plan‐specific OAR DVH points to generally compare dose sparing between modalities. Points of interest (POIs) specific to each extremity sarcoma plan include V40 (%) of the tibia, humerus, and right femur. POIs specific to each body sarcoma plan include $D_{\text{mean}}$ of the colon, $D_{\text{max}}$ of the right femur, V20 of the left lung, $D_{\text{max}}$ of the cauda equina, and $D_{\text{max}}$ of the rectum. The target coverage parameters HI and CI are defined as:

$$HI=\frac{D_{95}}{D_{5}};CI=\frac{Vol_{PTV_{Rx}}}{Vol_{PTV_{ALL}}}$$

where, for $HI,D_{5}$ is the dose to 5% of the PTV and $D_{95}$ is the dose to 95% of the PTV. For $CI,\;Vol_{PTVR_{x}}$ is the PTV volume receiving the prescribed dose and $Vol_{PTVALL}$ is the total patient volume receiving the prescribed dose. Both HI and CI are perfect at unity, and depreciate as they approach zero.

Treatment efficiency was compared by treatment time (net beam‐on time, in seconds) and monitor unit number for all plans. Plan quality assurance was evaluated with a 3%/3 mm gamma criteria using a Sun Nuclear ArcCHECK device. Current clinical standards require the global gamma passing rate percentage greater than 90% for each plan. All parameters were assessed for statistical significance between treatment modalities (VMAT‐MCO vs. IMRT‐MCO) by the two‐tailed Wilcoxon signed‐rank test.

## III. RESULTS

All 10 VMAT‐MCO generated treatments met physician‐imposed PTV coverage (100% of the PTV receiving more than 95% of prescription dose) and plan‐specific OAR dose‐sparing constraints. PTV homogeneity (HI=0.93±0.03 for VMAT‐MCO vs. 0.92±0.04 for IMRT‐MCO) and conformity (CI=0.91±0.09 for VMAT‐MCO vs. 0.89±0.08 for IMRT‐MCO) showed no significant differences between planning modalities. Skin dose, scaled to a PTV prescription dose of 5000 cGy, slightly decreased on average for VMAT‐MCO plans at D_50_ (340±570 vs. 350±500). A summary of target coverage and skin dose‐sparing comparisons is shown in Table 2.

**Figure 1:**
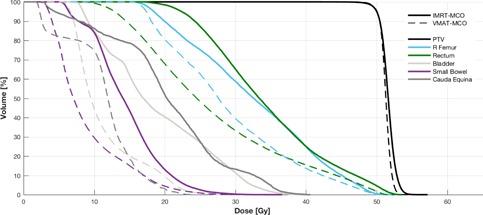
Dose distribution comparison for plan 9 (PTV right gluteus to 5000 cGy).

**Figure 2:**
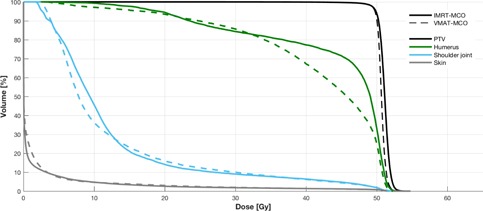
Dose distribution comparison for plan 3 (PTV right arm to 5000 cGy).

The wide spread of target location (e.g., right gluteus compared to right calf) within the patient cohort prevented additional baseline OAR dose‐sparing comparisons between modalities. To overcome this, we compared a relevant OAR DVH point for each plan pair, and we observed a general improvement for VMAT‐MCO over IMRT‐MCO plans, as seen in Table 3. VMAT‐MCO provided superior dose sparing to 9 of the 10 points of interest, with the most significant improvements found in plan 9 (cauda equina $D_{\text{max}}$ reduced from 4,048 cGy in IMRT‐MCO to 2,410 cGy in VMAT‐MCO) and plan 6 (colon $D_{\text{mean}}$ reduced from 1,779 cGy in IMRT‐MCO to 1,028 cGy in VMAT‐MCO). Figure 1 shows a dose distribution comparison between VMAT‐MCO and IMRT‐MCO for plan 9 (body sarcoma), which indicates the improvements in target coverage for the PTV (right gluteus) and OAR sparing for the cauda equina, rectum, right femur, bladder, and small bowel. This illustrates the general improvements made by VMAT‐MCO over IMRT‐MCO in terms of dose sparing. Figure 2 shows a dose distribution comparison between VMAT‐MCO and IMRT‐MCO for plan 3 (extremity sarcoma), which indicates the improvement in target coverage for the PTV (right arm) and OAR sparing for the humerus.

**Table 2:** Average plan results for IMRT‐MCO and VMAT‐MCO plans

*Index*	*IMRT‐MCO*	*VMAT‐MCO*	*Statistically significant?*
PTV HI	0.92±0.04	0.93±0.03	No
PTV CI	0.89±0.08	0.91±0.08	No
Skin $D_{\text{mean}}{\left(\text{cGy}\right)}^{a}$	990±590	1020±570	No
Skin $D_{50}{\left(\text{cGy}\right)}^{a}$	350±500	340±570	No
Delivery time	519±173	166±32	Yes (p<0.01)
Output	759±370	488±136	Yes (p<0.05)

a Scaled to Rx Dose of 5000 cGy.

**Table 3:** Selected DVH points of interest (POIs) for each plan

*Plan #*	*OAR*	*POI*	*IMRT‐MCO*	*VMAT‐MCO*
1	Tibia	V40	4.9%	4.2%
2	Humerus	V40	77.0%	67.0%
3	Humerus	V40	45.0%	47.0%
4	Tibia	V40	53.0%	50.0%
5	Rt femur	V40	26.0%	25.5%
6	Colon	$D_{\text{mean}}$	1779 cGy	1028 cGy
7	Rt femur	$D_{\text{max}}$	5149 cGy	5033 cGy
8	Lt lung	V20	3.1%	0.5%
9	Cauda equina	$D_{\text{max}}$	4048 cGy	2410 cGy
10	Rectum	$D_{\text{max}}$	1729 cGy	1636 cGy

**Figure 3:**
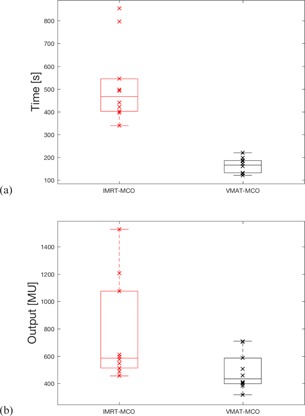
Delivery time (a) and plan MU (b) comparisons between VMAT‐MCO and IMRT‐MCO plans.

Plan delivery time showed statistically significant improvements of 213% on average for VMAT‐MCO plans (average delivery time of 166±32 s) as compared to IMRT‐MCO plans (average delivery time of 519±173 seconds), as illustrated in Fig. 3(a). Monitor unit number also showed statistically significant improvements for VMAT‐MCO plans (average of 488±570 monitor units) as compared to IMRT‐MCO (average of 759±370 monitor units), as seen in Fig. 3(b). VMAT‐MCO plan deliverability was confirmed and dosimetric measurements showed a slight improvement in gamma passing rate average for VMAT‐MCO (99.4%±0.9%) as compared to IMRT‐MCO (98.7%±1.6%). Individual plan results for plan quality and treatment efficiency can be found in Tables 4 and 5, respectively.

**Table 4:** Overview of homogeneity, conformity, and dose‐sparing comparisons between VMAT‐MCO and IMRT‐MCO plans

	*IMRT‐MCO*				*VMAT‐MCO*			
*Plan #*	*HI*	*CI*	$D_{mean}$ *(cGy)*	$D_{50}$ *(cGy)*	*HI*	*CI*	$D_{mean}$ *(cGy)*	$D_{50}$ *(cGy)*
1	0.94	0.97	1939	1512	0.94	0.98	1989	1656
2	0.93	0.96	710	24	0.93	0.94	710	28
3	0.96	0.89	372	32	0.96	0.92	196	4
4	0.84	0.82	848	23	0.91	0.89	776	25
5	0.89	0.96	256	2	0.91	0.96	1072	52
6	0.92	0.96	1429	395	0.93	0.99	1282	190
7	0.95	0.84	1407	444	0.96	0.81	1419	438
8	0.94	0.92	621	43	0.94	0.97	655	133
9	0.86	0.71	1599	635	0.86	0.74	1494	475
10	0.93	0.86	399	109	0.94	0.87	285	73
Mean	0.92±0.04	0.89±0.08	–	–	0.93±0.03	0.91±0.08	–	–

**Table 5:** Comparison of delivery time and output for IMRT‐MCO and VMAT‐MCO

	*IMRT‐MCO*		*VMAT‐MCO*	
*Plan*	*Time (s)*	*Output (MU)*	*Time (s)*	*Output (MU)*
1	497	595	160	384
2	339	576	121	317
3	421	455	160	409
4	441	487	132	398
5	493	547	130	406
6	402	612	198	709
7	545	513	220	418
8	396	1075	184	586
9	797	1206	172	506
10	854	1527	186	704
Mean	519±173	759±370	166±32	488±136

## IV. DISCUSSION

We successfully generated a set of 10 clinical VMAT‐MCO sarcoma plans, and subsequently evaluated and compared the cohort to paired IMRT‐MCO plans in terms of plan quality and treatment efficiency. The optimization and individualization afforded by MCO provides high‐quality IMRT sarcoma plans, and it is vital to confirm that plan quality is maintained using VMAT‐MCO before transitioning to a VMAT‐MCO standard in the clinic. This study evaluated the VMAT‐MCO algorithm for the complex sarcoma site; to our knowledge VMAT‐MCO has only been studied dosimetrically for prostate. Non‐MCO VMAT has been tested and commissioned on complex sites for other TPS (e.g., Varian RapidArc), but inherent algorithmic and delivery differences between systems require a VMAT‐MCO‐specific validation in order to proceed with necessary clinical confidence in treatment. This same degree of confidence was required for MCO‐IMRT studies.

In theory, the increase in gantry angle freedom for VMAT compared to IMRT allows for a more uniform spread of skin dose, and improved dose conformity and homogeneity in some cases. In this study we confirm VMAT‐MCO has comparable plan quality and OAR sparing to IMRT‐MCO for sarcoma plans. We found dose sparing to the skin to be improved in VMAT‐MCO plans, and target coverage was maintained and at times improved. Dose sparing to other OARs could not be directly intercompared between modalities for statistical significance due to the variation of relevant structures by plan. VMAT‐MCO body sarcoma treatments showed more pronounced improvements in dose sparing over IMRT‐MCO treatments in the body sarcoma subset, which is due to the higher degree of OAR overlap in more centrally located targets. For example, the more uniform distribution of dose permitted by VMAT's increased gantry degree freedom allowed for significantly improved sparing in V20 for the left lung in plan 8; the corresponding IMRT plan solution space was confined to only eight gantry angles which required depositing a higher dose to a larger volume of the proximal lung in order to achieve prescription dose in the PTV.

While the actual patient benefit of homogeneity and conformity remains largely unproven,^(17)^ it was an objective in both IMRT‐MCO and VMAT‐MCO optimization, and thus represents a relevant comparison metric. The most significant improvements made using VMAT‐MCO are the decreases in monitor units (p<0.05) and treatment delivery time (p<0.01) which are made possible by the reduced “beam‐on” time of VMAT as compared to IMRT. Furthermore, the dynamic nature of VMAT delivery helps overcome the trade‐off between treatment time and treatment plan quality which can impede IMRT treatment efficiency.^(18)^ The decrease in monitor units per patient plan for VMAT extends linac life span, conserves energy, and has been shown to reduce the likelihood of secondary cancers.^(19)^ The decrease in delivery time minimizes patient discomfort, reduces the chances of patient movement during treatment, and allows for more patients to be treated.

We tested the gamma passing rate in order to confirm quality assurance for each plan. While VMAT shows slightly improved results over IMRT, the uncertainty of the metrics’ utility disqualifies it from being used conclusively in intercomparison.

RayStation has resolved issues with full‐fledged implementation of MCO for VMAT planning, which permits the extension of VMAT‐MCO planning to complex treatment sites. We first examined VMAT‐MCO for sarcomas and plan to undergo a similarly exhaustive commissioning process on other sites. The exciting developments made in VMAT‐MCO require detailed, site‐specific commissioning, and we successfully validated the clinical feasibility and utility of VMAT‐MCO in lieu of IMRT‐MCO. The results of this study confirm the improvements in treatment efficiency and dosimetric quality afforded by VMAT‐MCO planning for complex treatment sites.

## V. CONCLUSION

We compared dosimetric quality and treatment efficiency between the VMAT‐MCO and IMRT‐MCO planning modules in RayStation for 10 sarcoma cancer patients receiving standard fractionation treatment. This study highlights the advantages in treatment efficiency and uncompromised plan quality made possible by VMAT‐MCO as compared to IMRT‐MCO.

## COPYRIGHT

This work is licensed under a Creative Commons Attribution 3.0 Unported License.
